# Medication Adherence and Belief about Medication among Vietnamese Patients with Chronic Cardiovascular Diseases within the Context of Implementing Measures to Prevent COVID-19

**DOI:** 10.3390/jcdd9070202

**Published:** 2022-06-26

**Authors:** Nguyet Kim Nguyen, Han Gia Diep, Hung Huynh Vinh Ly, Ngoc Le Minh Nguyen, Katja Taxis, Suol Thanh Pham, Trang Huynh Vo, Thang Nguyen

**Affiliations:** 1 Department of Pharmacology and Clinical Pharmacy, Can Tho University of Medicine and Pharmacy, Can Tho City 900000, Vietnam; kimnguyetnguyen@gmail.com (N.K.N.); dgiahan6002@gmail.com (H.G.D.); ptsuol@ctump.edu.vn (S.T.P.); 2 Faculty of Medicine, Can Tho University of Medicine and Pharmacy, Can Tho City 900000, Vietnam; 1853010822@student.ctump.edu.vn; 3 Department of Traditional Medicine, Can Tho University of Medicine and Pharmacy, Can Tho City 900000, Vietnam; minhngoc101001@gmail.com; 4 Groningen Research Institute of Pharmacy, University of Groningen, 9713 AV Groningen, The Netherlands; k.taxis@rug.nl

**Keywords:** medication adherence, beliefs about medication, chronic cardiovascular diseases, COVID-19, Vietnamese

## Abstract

Background: Long-term adherence is crucial for optimal treatment outcomes in chronic cardiovascular diseases (CVDs), especially throughout the COVID-19 wide-spreading periods, making patients with chronic CVDs vulnerable subjects. Aim: To investigate the relationship between the characteristics, beliefs about prescribed medication, COVID-19 prevention measures, and medication adherence among patients with chronic CVDs. Methods: This is a cross-sectional study of outpatients with chronic CVDs in Southern Vietnam. The specific parts regarding the Beliefs about Medicines Questionnaires (BMQ—Specific) and the General Medication Adherence Scale (GMAS) were applied to assess the beliefs about and adherence to medication. The implementation measures to prevent COVID-19 in patients were evaluated according to the 5K message (facemask, disinfection, distance, no gathering, and health declaration) of the Vietnam Ministry of Health. A multivariable logistic regression with the Backward elimination (Wald) method was used to identify the associated factors of medication adherence. Results: A slightly higher score in BMQ-Necessity compared to BMQ-Concerns was observed. A total of 40.7% of patients were recorded as having not adhered to their medications. Patients’ behavior was most frequently self-reported by explaining their non-adherence (34.7%). Statistical associations were found between rural living place, unemployment status, no or only one measure(s) of COVID-19 prevention application, and medication adherence. Conclusion: During the COVID-19 spreading stage, patients generally showed a positive belief about medication when they rated the importance of taking it higher than its side effects. The data analysis suggested that rather than patients’ beliefs, the clinicians should consider the patient factors, including living place, employment, and the number of epidemic preventive measures applied for guiding the target patients for improving medication adherence.

## 1. Introduction

Cardiovascular diseases (CVDs) are the leading causes of death globally, accounting for about 17.9 million (32%) deaths in 2019 [1]. By 2030, the number of CVD deaths is estimated to rise to approximately 23.3 million [2]. Over three-fourths of CVD deaths occur in low- and middle-income nations, including Vietnam [1]. In the pace of coronavirus disease-2019 (COVID-19), healthcare delivery for non-communicable diseases has been severely disrupted due to the fear of contagion, government-issued social distancing rules, and travel limitations [3]. The frequency of emergency department visits by patients with chronic CVDs has decreased considerably [4]. This is a potentially significant problem because patients with chronic CVDs require frequent revisits, follow-ups, check-ups, and prescription refills. Furthermore, among patients with severe symptoms of COVID-19, 58% had hypertension, 25% had heart disease, and 44% had arrhythmia, indicating that CVD patients are vulnerable subjects in need of attention during the pandemic [5].

Long-term adherence patterns are critical for optimal treatment outcomes in CVDs and must be highlighted, especially during the COVID-19 wide-spreading periods. Medication adherence refers to how patients behave according to healthcare providers’ recommendations [6]. It is considered "good" when 80 percent or more of recommendations are adhered to by patients [7]. In numerous studies, high adherence to antihypertensive medication treatment has been linked to improved blood pressure (BP) control and a lower risk of CVDs [8,9]. Patients with poor drug adherence have a higher chance of bad outcomes and higher healthcare expenses resulting from cardiovascular complications than patients with good adherence [10]. However, the full extent of the benefits of medication adherence in patients with chronic CVDs is often unidentified due to the low prevalence of medication adherence. Only 55.5% of chronic-condition patients reported adhering to medication [11]. Understanding the new and emerging factors that influence adherence could aid in developing the intervention, timely lifestyle counseling, and treatment adjustments to improve medication adherence. Obstructions to medication adherence comprise patients’ beliefs about treatment, expenses, lack of health insurance, comorbidities, and polypharmacy [12,13]. However, the obstacles to medication adherence vary in different regions and periods, especially in light of the new circumstances where the COVID-19 outbreak may affect medication adherence. 

Non-adherence could be unintentional (patients’ forgetfulness or inability to handle or afford the medication) or intentional (more frequently, the result of a conscious decision) [14]. Intentional non-adherence is motivated by personal beliefs about the disease’s potential dangers, treatment risks, and perceived treatment needs [15]. The adherence is derived from a patient deciding that the treatment needs exceed any worries about the therapy’s possible adverse effects. The BMQ-Specific is a widely used assessment of beliefs about medication, evaluating both opinions about the need for a specific drug for a specific disease (Specific Necessity) and concerns about the treatment’s potential adverse effects (Specific Concerns). Appropriate medication beliefs play a significant role in treatment, especially during the COVID-19 outbreak and social restrictions. Patients with poor knowledge might also have poor adherence to COVID-19 prevention measures and CVD medication during the COVID-19 outbreak, making them significant vulnerable subjects. This study aimed to investigate the relationship between patients’ characteristics, attitudes regarding CVD medication, adoption of COVID-19 prevention measures, and adherence to medication; thus, it is a timely assessment of adherence obstructions and the provision of feasible healthcare interventions for patients with chronic CVDs.

## 2. Materials and Methods

### 2.1. Study Design and Setting

We conducted a cross-sectional study of patients with chronic cardiovascular diseases in some Southern Vietnam provinces’ outpatient clinics, comprising Can Tho, Ca Mau, Tay Ninh, Kontum, and Kien Giang, from March to May 2021. The locations were the workplaces of some students at Can Tho University of Medicine and Pharmacy, who were previously trained and were in charge of collecting data. 

### 2.2. Participants

We used a convenient sampling method to recruit participants. Eligible patients had confirmed cardiovascular diseases with at least 6 months of treatment and had follow-ups during office hours.

The sample size was estimated using the formula for a single proportion with an estimated adherence proportion of 49.8%, an assumed margin of error of 4%, and a confidence level of 95% [16]. An additional 20% were selected to minimize the error, whereby at least 720 participants were needed. 

### 2.3. Data Measurements

We conducted a face-to-face interview with eligible patients visiting the outpatient clinics during research time (office hours). The interview content was based on the prepared questionnaire, including patients’ general information, General Medication Adherence Scale (GMAS), and the Beliefs about Medicine Questionnaire (BMQ).

Patients’ general information included demographic characteristics (age, sex, living place, employment, education level, monthly income), comorbidities, disease duration, and treatment.

Patients’ adherence to medication was evaluated by GMAS, which was previously adjusted, and validated in Vietnam. The scale included 11 questions, each with four possible answers (always–mostly–sometimes–never, scoring from 3 to 0, respectively), which measured adherence in three different aspects of patients’ behavior, medication burden, and cost-related burden. The total score categorizes medication adherence from high to poor, with 0–26 points for non-adherence and 27–33 points for adherence. We assessed the medication adherence proportion by the ratio of adherence patients to the total number of patients.

Patients’ beliefs about medication related to cardiovascular diseases were evaluated by BMQ–Specific. The questionnaire includes two subscales: BMQ–Necessity (5 statements) and BMQ–Concerns (5 statements), each item of which scores from 1 (strongly disagree) to 5 (strongly agree) on a 5-point Likert scale. The total BMQ–N and BMQ–C scores range from 5 to 25. Higher BMQ–N scores indicate a stronger belief in the medication prescribed to sustain health, while higher BMQ–C scores indicate more significant concern about the medication’s potential side effects.

We recorded patient applications of measures to prevent COVID-19 during the medical visits according to the 5K-message (wearing a facemask, disinfecting, keeping distance, not gathering, and health declaration) recommended by the Ministry of Health of Vietnam. The number of measures applied (0–1 measure and ≥2 measures) was evaluated to determine whether it was associated with patient medication adherence or not.

The primary outcome was the prevalence of patients’ adherence. The secondary outcomes were relationships between population characteristics, measures to prevent the COVID-19 pandemic, medication beliefs, and adherence.

### 2.4. Ethics Approval

Ethical approval for this study was obtained from the Medical Ethics Councils of Can Tho University of Medicine and Pharmacy and the provincial hospital (1541/QD-DHYDCT). Participants were informed of the purpose and procedure of the study and voluntarily signed an informed consent form.

### 2.5. Statistical Methods

Qualitative variables were presented by proportions and frequencies, and quantitative variables were described by means ± standard deviations. We modeled a multivariable logistic regression analysis assuming the non-adherence to medication as the outcome variable and population characteristics, patient’s belief about medication, and the number of measures applied to prevent the Covid-19 pandemic as explanatory ones. The variables with univariable results with *p*-values ≤ 0.1 were analyzed by multivariable logistic regression with the Backward elimination (Wald) method with odds ratios (OR) and 95% confidence intervals (CIs). The *p*-values < 0.05 were considered statistically significant. Data were analyzed through SPSS 22.0.

## 3. Results

### 3.1. Population Characteristics

The study included 1038 patients (female = 587; 56.6%, mean age = 62.7 ± 11.84), of whom 84.2% were unemployed (retired, were running their own business, etc., without being paid), and 70.5% had less than one comorbidity. The participants had an average of 8.45 ± 6.46 years of disease and 8.22 ± 6.40 years of treatment (Table 1). 

### 3.2. Evaluation of the Beliefs of Patients with Cardiovascular Diseases about Medication

The mean scores of BMQ-N and BMQ-C were 19.1 ± 3.374 and 17.48 ± 3.226, indicating relatively positive medication beliefs (Table 2).

### 3.3. Medication Adherence

The adherence proportion among patients with chronic CVDs was relatively low (59.3%) (Table 3). Behavior was the most common reason for non-adherence (34.3%) (Table 4).

### 3.4. Attitudes and Practices of Patients in the COVID-19 Pandemic Period

The most common preventive measures were wearing a facemask (95.5%) and disinfecting (47.3%). Regarding the combination of measures, the proportion of combining more than two measures was 56.6% (Table 5).

### 3.5. Evaluation of the Relationship between Patient Characteristics, Medication Beliefs, Numbers of 5K Measures Applied, and Patient Adherence

A multivariable logistic regression was applied for factors related to patient characteristics, medication beliefs, and numbers of measures applied, of which *p*-values of the simple regression analysis were ≤0.1. Patients living in rural areas (OR = 1.375; 95% CI = 1.038–1.822; *p* = 0.027), that were unemployed (OR = 1.535; 95% CI = 1.012–2.331; *p* = 0.044), and that applied 0–1 measure (OR = 1.660; 95% CI = 1.276–2.161; *p* < 0.001) had better adherence to medication (Table 6).

## 4. Discussion

The study results indicated that a positive belief about the prescribed medication for patients with chronic CVDs tended to be more beneficial for their condition (19.1 ± 3.374) than detrimental (17.48 ± 3.226). In contrast, healthcare providers should pay attention to a relatively low level of medication adherence in this population. Although most patients reported that they applied the COVID-19 pandemic prevention measures, particularly wearing a facemask (95.5%) during medical visits, the majority of patients reported that they did not simultaneously apply multiple measures according to the 5K messages (43.4%). It is also noteworthy that strategies to enhance cardiovascular medication adherence should target the unemployed living in rural areas. Applying one or none of the preventive measures is possibly a sign of poorer adherence in patients with chronic CVDs.

We collected data through face-to-face interviews with chronic patients coming to the cardiology clinic for follow-up visits within a background context where social distancing and lockdown orders were not stringently implemented. This method creates conveniences for the patient and is suitable for elderly patients who do not often access means of communication such as the telephone or internet, thus providing easy access to the target population. This method distinguishes our study from other studies studying the same topic during the COVID-19 epidemic that used the internet or telephone for data collection.

During the COVID-19 pandemic, chronic patients have several barriers to adhering to CVD medication. Patients’ belief about medication is frequently considered a critical factor underlying the non-adherence to medication [14,17,18,19,20] This study explored this feature as a potential factor associated with medication adherence in patients with chronic CVD. The BMQ-Specific helped assess patients’ beliefs about medication for personal use. The BMQ-Necessity score in our study with an average of 19.1, which is higher than that of Sjölander et al. (score of 18.87) [14] and Bawab et al. (score of 18.36) [18] but lower than several studies during the pre-pandemic [17,19] and the epidemic periods [17,20], ranging from scores of 20 to 21.5. Our study’s average BMQ-Concerns score of 17.48 was higher than most recent studies fluctuating from 12.25 to 17 [14,17,18,19,20] but was significantly lower than that reported by Olorunfemi et al. [17]. In general, patients with chronic CVDs in our study rated their need for medication as greater than their concern about the adverse effects of the medicines prescribed. Therefore, patients are more inclined to accept the medication, a positive sign which was reported in Part et al.’s study [21].

From the univariable regression model, it is evident that patients’ beliefs about medication did not statistically appear to influence the medication adherence in patients with chronic CVDs during the COVID-19 spreading period in Vietnam. This is, surprisingly, contrary to the findings of several prior studies recording the relation between patients’ medication-related beliefs and medication adherence during the pre-epidemic [14,17,18,19] and during the epidemic period [20]. One possible explanation for this difference may be that patients in our study generally had a positive belief about medication with a higher mean score in BMQ-Necessity than BMQ-Concerns; thus, patients’ beliefs about medication were not likely to be the main reason for patients not taking their prescribed medicine.

Compared with other studies in chronic patients during non-COVID-19 periods, patients’ adherence in this study was relatively low. Nguyen et al. have recorded a high medication adherence in heart failure patients (mean = 4.01, SD = 0.77), while Truong and Nguyen reported that 68.1% of type 2 diabetes outpatients adhered to their medication [22,23]. Upon the occurrence of the epidemic, there seemed to be a variation in the results of studies assessing medication adherence. On the other hand, the percentage of patients’ adherence in this study was slightly higher than in the previous study by Nguyen et al. in hypertensive patients in Vietnam rural, with 49.8% in the pre-epidemic period [16]. This may be due to the different population characteristics between the two studies, in which the location factor (rural or urban) was shown as an association with medication adherence [24,25,26]. In developing countries, Simon et al. recently observed less than 50% of chronic patients adhered to cardiovascular medication [27]. In Ethiopia, a study by Shimels et al. reported an even lower figure for diabetic and hypertensive patients during the COVID-19 epidemic with only 28% adherence [28]. In contrast, the percentage of patients’ adherence in our study was lower than in an earlier study in Iraq that determined adherence based on a questionnaire (81.8%) [29]. However, most published studies on medication adherence in CVD patients used the eight-item Morisky Medication Adherence Scale (MMAS-8) questionnaire, which is distinct from the GMAS questionnaire we used since it does not mention the economic factors affecting adherence. In addition, the COVID-19 situation in different countries and periods may affect drug adherence rates.

Investigating the causes of poor adherence supports healthcare professionals’ ability to make appropriate interventions. About 1/3 of patients reported that they failed to remember to take their medicines and stopped using the medication because they had a low perceived need for treatment and/ or arbitrarily changed the usage. Forgetfulness and low perceived need for treatment were the most common reasons for patients’ previously reported non-adherence [16,30,31]. However, the unavailability of medication and financial difficulties are the most common causes reported during the COVID-19 epidemic in recent studies [28,32]. This difference is that the COVID-19 epidemic in Vietnam was in a spreading stage of the epidemic at the time of the study. The economic impacts were not noticeable, and lockdown measures had not been applied.

Our study was conducted when Vietnam experienced three waves of COVID-19 from early 2020 to early 2021 and when the 4th wave was initially spreading in the community in some major cities. Before this study, the Ministry of Health of Vietnam issued recommendations on COVID-19 epidemic prevention measures for people participating in public gatherings/activities. It disseminated the 5K message on the media to raise the highest awareness and ensure community compliance. Patients’ adoption of preventative measures were behaviors that presented their understanding of the epidemic and their self-discipline during direct follow-up visits at healthcare facilities. Implementing 5K measures in patients with chronic CVD has not been reported since the Vietnamese Ministry of Health issued the 5K messages. Although all five measures are recommended for co-application to enhance the best prevention, depending on the individual, some measures might not be applied as people do not know about the measures or forget them; this is especially relevant in elderly patients. This could be the possible reason for about 43.4% of patients taking only one or no protective measures besides patients’ awareness and reasoning. Our result is consistent with Dorfman et al.’s finding that additional protective measures, including the avoidance of school, voluntary lockdowns, frequent disinfection, etc., were taken by almost 50% of chronic patients [33]. However, the implementation rate in our study was lower than that of Nguyen et al., which reported a relatively high adherence rate to specific measures such as wearing a mask, disinfecting, and social distancing (mostly over 80%). This is possible because Nguyen et al. studied a generally young population with an average age of 30 years and used yes/no questions to assess the adherence rate [34].

Our study determined many factors possibly related to medication non-adherence through the univariable regression model. Except for sex and comorbidities, most factors related to patient characteristics were associated with patients’ adherence. These associations reveal that a low quality of life, relating to factors such as income, employment, and place of living, could interfere with patients’ adherence to medication. In many cases, these associations can also be explained by the fact that high-income patients living in urban areas tend to have higher levels of education. Moreover, educational level is strongly associated with medication adherence, as reported elsewhere [35,36,37]. Longer disease and treatment durations significantly interfered with patients’ adherence to medication, which is consistent with previous studies [24,38,39]. However, we did not find an association between the number of comorbidities and medication adherence, contrary to findings previously reported [28,40,41].

In the final logistic regression model with the Backward elimination (Wald) method, the number of patients’ measures presented a significant association with medication adherence. This could be because medication adherence is partly explained by patients’ awareness of their health status. In other words, the more patients were aware of preventive measures and proactively complied with the 5K messages, the more likely they had higher medication adherence levels.

The ongoing COVID-19 pandemic significantly impacted the management of patients with chronic diseases. Telehealth and telemedicine are considered inevitable during the COVID-19 outbreak [32,42,43,44,45]. However, Vietnam’s healthcare system, which is accustomed to conventional face-to-face medical management with limited resources, may face many obstacles while shifting toward telehealth and telemedicine. By assessing patients’ beliefs, medication adherence, 5K message implementation, and identifying factors associated with adherence in chronic patients with chronic CVD, we presented a relatively comprehensive view of patients’ adherence to medication under the spread of COVID-19 in Vietnam. Our results support the elaboration of defining health care for chronic patients with chronic CVDs and help to prepare for the potential shift toward types of telehealth in the healthcare system to cope with new situations in the current outbreak of COVID-19. 

However, the findings in our study should be interpreted with some caution due to COVID-19-related uncertainty surrounding clinical practitioners and patients over the last two years, which might have influenced the observed results. Since the study was conducted in an early spreading stage of the COVID-19 epidemic in Vietnam, it is difficult to predict whether the same results will be observed in other periods such as a breakout or post-COVID-19, which could have varying degrees of impact on healthcare systems and patient behaviors. Therefore, further studies in different periods of the COVID-19 pandemic are necessary to investigate the factors related to patients’ adherence, thus providing appropriate and timely interventions. Along with this limitation, the face-to-face interview method of data collection while minimizing social contact might have contributed to the poor quality of the patients’ responses, whereby the patients were inclined to answer as quickly as possible or refuse to be interviewed. To prevent this bias, future research should emphasize the importance of the answer quality to participants by employing assistants during the data collection and providing clear instructions in the questionnaire, thus ensuring sufficient access to the target population. Patients’ prior beliefs regarding medication may have influenced their willingness to vaccinate. Further studies based on the results of this study could study the effects of prior beliefs on the willingness to vaccinate against COVID-19; thus, such a study could support appropriate interventions to improve the injection rate and alleviate the pandemic.

## 5. Conclusions

In the context of the COVID-19 epidemic spreading in Vietnam, over 40% of chronic cardiovascular patients did not adhere to their prescribed medication. One-third of non-adherent patients self-reported that their behaviors were the cause of the non-adherence. On the other hand, the BMQ score in the study patients reflected a positive belief about the importance of taking the medicines rather than worrying about the side effects. However, we did not find a relationship between patients’ beliefs and medication non-adherence using univariate regression models. In contrast, the application of 0 or only 1 COVID-19 prevention measure was significantly associated with poor medication adherence, along with two other factors: living in rural areas and not working for a company or organization. Thus, clinicians need to focus on patients with these characteristics to improve medication adherence, thereby improving the effectiveness of treatment.

## Figures and Tables

**Table 1:** Characteristics of the study population (N = 1038).

Characteristics		Frequency (*n*)	Percentage (%)
Age Mean ± standard deviation (years old): 62.7 ± 11.84	< 60 years old	391	37.7
	≥ 60 years old	647	62.3
Sex	Male	451	43.4
	Female	587	56.6
Living place	Rural areas	561	54.0
	Urban areas	477	46.0
Employment	Employed ^a^	164	15.8
	Unemployed ^b^	874	84.2
Level of education	Illiterate/Elementary	368	35.5
	High school or higher	670	64.5
Monthly income	≤5 million VND	607	58.5
	>5 million VND	431	41.5
Duration of disease Mean ± standard deviation (years): 8.45 ± 6.46	≤5 years	424	40.8
	>5 years	614	59.2
Duration of treatment Mean ± standard deviation (years): 8.22 ± 6.40	≤5 years	447	43.1
	>5 years	591	56.9
Comorbidities Mean ± standard deviation: 1.15 ± 0.98	0–1 comorbidity	732	70.5
	≥2 comorbidities	306	29.5

^a^ Being paid to work for a company or organization. ^b^ Retired, running own business, etc., without being paid.

**Table 2:** Evaluation of the beliefs of patients with cardiovascular disease with drugs based on the BMQ scale (N = 1038).

	Totally Agree *n* (%)	Agree *n* (%)	Uncertain *n* (%)	Disagree *n* (%)	Totally Disagree *n* (%)	Mean Score (SD)
Specific-Necessity (BMQ-N) ^a^						19.1 ± 3.374
(N) My health, at present, depends on my medicines	177 (17.1)	579 (55.8)	174 (16.8)	81 (7.8)	27 (2.6)	3.77 ± 0.912
(N) My life would be impossible without my medicines	168 (16.2)	559 (53.9)	194 (18.7)	102 (9.8)	15 (1.4)	3.74 ± 0.896
(N) Without my medicines I would be very ill	159 (15.3)	565 (54.4)	220 (21.2)	83 (8.0)	11 (1.1)	3.75 ± 0.848
(N) My health in the future will depend on my medicines	195 (18.8)	585 (56.4)	175 (16.9)	62 (6.0)	21 (2.0)	3.84 ± 0.867
(N) My medicines protect me from my condition worsening	210 (20.2)	657 (63.3)	145 (14.0)	22 (2.1)	4 (0.4)	4.01 ± 0.68
Specific-Concerns (BMQ-C) ^b^						17.48 ± 3.226
(C) Having to take medicines worries me	93 (9.0)	411 (39.6)	275 (26.5)	232 (22.4)	27 (2.6)	3.3 ± 0.997
(C) I sometimes worry about the long-term effects of my medicines	141 (13.6)	587 (56.6)	213 (20.5)	88 (8.5)	9 (0.9)	3.74 ± 0.83
(C) My medicines are a mystery to me	96 (9.2)	483 (46.5)	260 (25.0)	181 (17.4)	18 (1.7)	3.44 ± 0.941
(C) My medicines disrupt my life	85 (8.2)	500 (48.2)	217 (20.9)	214 (20.6)	22 (2.1)	3.4 ± 0.971
(C) I sometimes worry about becoming too dependent on my medicines	122 (11.8)	564 (54.3)	199 (19.2)	130 (12.5)	23 (2.2)	3.61 ± 0.926

^a^ Beliefs About Medicines Questionnaire Specific Section—Necessity Scale. ^b^ Beliefs About Medicines Questionnaire Specific Section—Concerns Scale.

**Table 3:** Medication adherence of patients with cardiovascular disease (N = 1038).

	Frequency (*n*)	Percentage (%)
Adherence	616	59.3
Non-adherence	422	40.7

**Table 4:** Characteristics of medication adherence based on GMAS scale (N = 1038).

Characteristics of Medication Adherence	Medication Adherence Level	
	Adherence *n* (%)	Non-Adherence *n* (%)
Non-adherence due to patient behavior (*)	682 (65.7)	356 (34.3)
Non-adherence due to additional disease and pill burden	837 (80.6)	201 (19.4)
Non-adherence due to financial constraints	813 (78.3)	225 (21.7)

(*): forget to take, stop taking, or arbitrarily change the dosage.

**Table 5:** Attitudes and practices of patients in COVID-19 pandemic (N = 1038).

Variables	Frequency (*n*)	Percentage (%)
Prevention		
Wearing facemask	991	95.5
Disinfection	491	47.3
Distance	195	18.8
Not gathering	149	14.4
Health declaration	186	17.9
Number of measures were applied		
0–1 measure	451	43.4
≥2 measures	587	56.6

**Table 6:** Univariable and multivariable regression analyses of the relationship between patient characteristics, medication beliefs, numbers of measures applied, and adherence.

Factors Affecting		Adherence Level		Univariable Logistic Regression		Multivariable Logistic Regression	
		Adherence *n* (%)	Non-Adherence *n* (%)	OR (95% CI)	*p*	OR (95% CI)	*p*
Patient characteristics							
Age	<60 years old	258 (66.0)	133 (34.0)	1	-	1	-
	≥60 years old	358 (55.3)	289 (44.7)	1.566 (1.207–2.032)	0.001	1.174 (0.869–1.585)	0.297
Sex	Male	281 (62.3)	170 (37.7)	1	-	1	-
	Female	335 (57.1)	252 (42.9)	1.243 (0.967–1.598)	0.089	1.182 (0.908–1.539)	0.213
Living place	Rural areas	302 (53.8)	259 (46.2)	1.652 (1.284–2.125)	<0.001	1.375 (1.038–1.822)	0.027
	Urban areas	314 (65.8)	163 (34.2)	1	-	1	-
Employment	Employed ^a^	121 (73.8)	43 (26.2)	1	-	1	-
	Unemployed ^b^	495 (56.6)	379 (43.4)	2.155 (1.484–3.128)	<0.001	1.535 (1.012–2.331)	0.044
Level of education	Illiterate/Elementary	188 (51.1)	180 (48.9)	1.693 (1.308–2.192)	<0.001	1.065 (0.780–1.445)	0.692
	High school or higher	428 (63.9)	242 (36.1)	1	-	1	-
Monthly income	≤5 million VND	331 (54.5)	276 (45.5)	1.628 (1.261–2.102)	<0.001	1.123 (0.830–1.520)	0.451
	>5 million VND	285 (66.1)	146 (33.9)	1	-	1	-
Comorbidities	0–1 comorbidity	443 (60.5)	289 (39.5)	1	-	-	-
	≥2 comorbidities	173 (56.5)	133 (43.5)	1.178 (0.899–1.544)	0.234	-	-
Duration of disease	≤5 years	276 (65.1)	148 (34.9)	1	-	1	-
	>5 years	340 (55.4)	274 (44.6)	1.503 (1.164–1.940)	0.002	2.184 (0.936–5.096)	0.071
Duration of treatment	≤5 years	285 (63.8)	162 (36.2)	1	-	1	-
	>5 years	331 (56.0)	260 (44.0)	1.382 (1.074–1.778)	0.012	0.559 (0.242–1.292)	0.174
Belief about medication ^c^							
Patients with positive belief		433 (59.2)	299 (40.8)	1	-	-	-
Patients with negative belief		183 (59.8)	123 (40.2)	0.973 (0.742–1.278)	0.846	-	-
Numbers of measures applied ^d^							
0–1 measure		230 (51.0)	221 (49.0)	1.845 (1.435–2.372)	<0.001	1.660 (1.276–2.161)	<0.001
≥2 measures		386 (65.8)	201 (34.2)	1	-	1	-

^a^ Being paid to work for a company or organization. ^b^ Retired, running own business, etc., without being paid. ^c^ Patients belief about medication is positive when the BMQ-Necessity score is equal to or higher than BMQ-Concerns, and vice versa for patients with negative beliefs.^d^ The number of COVID-19 pandemic prevention measures that the patient applied.

## Data Availability

The datasets generated during and/or analyzed during the current study are available from the corresponding author on reasonable request.
